# Supplementary figures and images for: Systematic Evaluation of Signal Peptide-Driven Protein Secretion in the Fast-Growing Cyanobacterium Synechococcus sp. PCC 11901
Source: Biomolecules. 2026 Jun 13;16(6):870. doi: 10.3390/biom16060870 (PMC13297417; doi:10.3390/biom16060870)

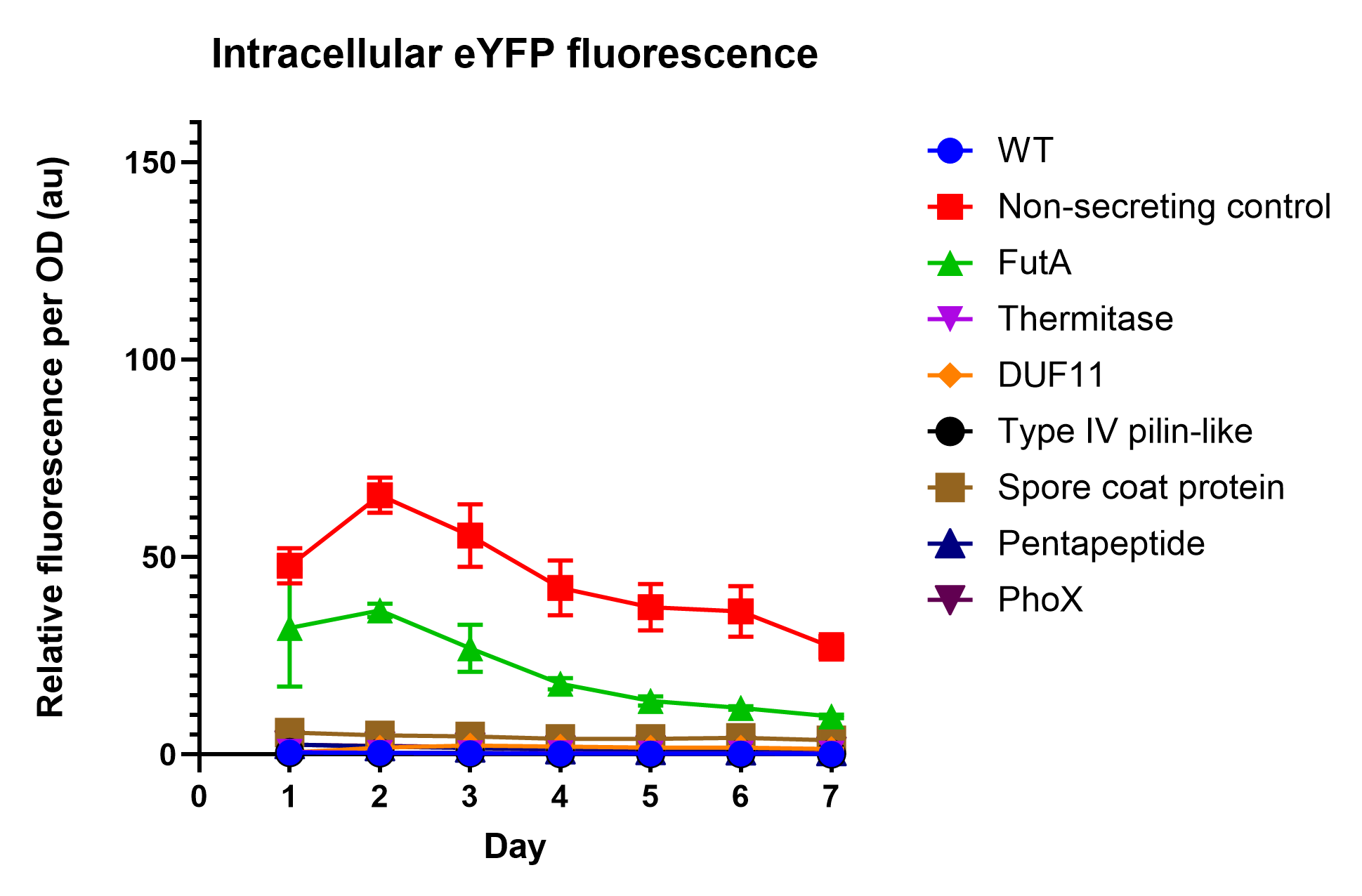

Supplement: Supplementary file 1 [file biomolecules-16-00870-s001.zip › Figure S1 Intracellular eYFP fluorescence time course.tif]

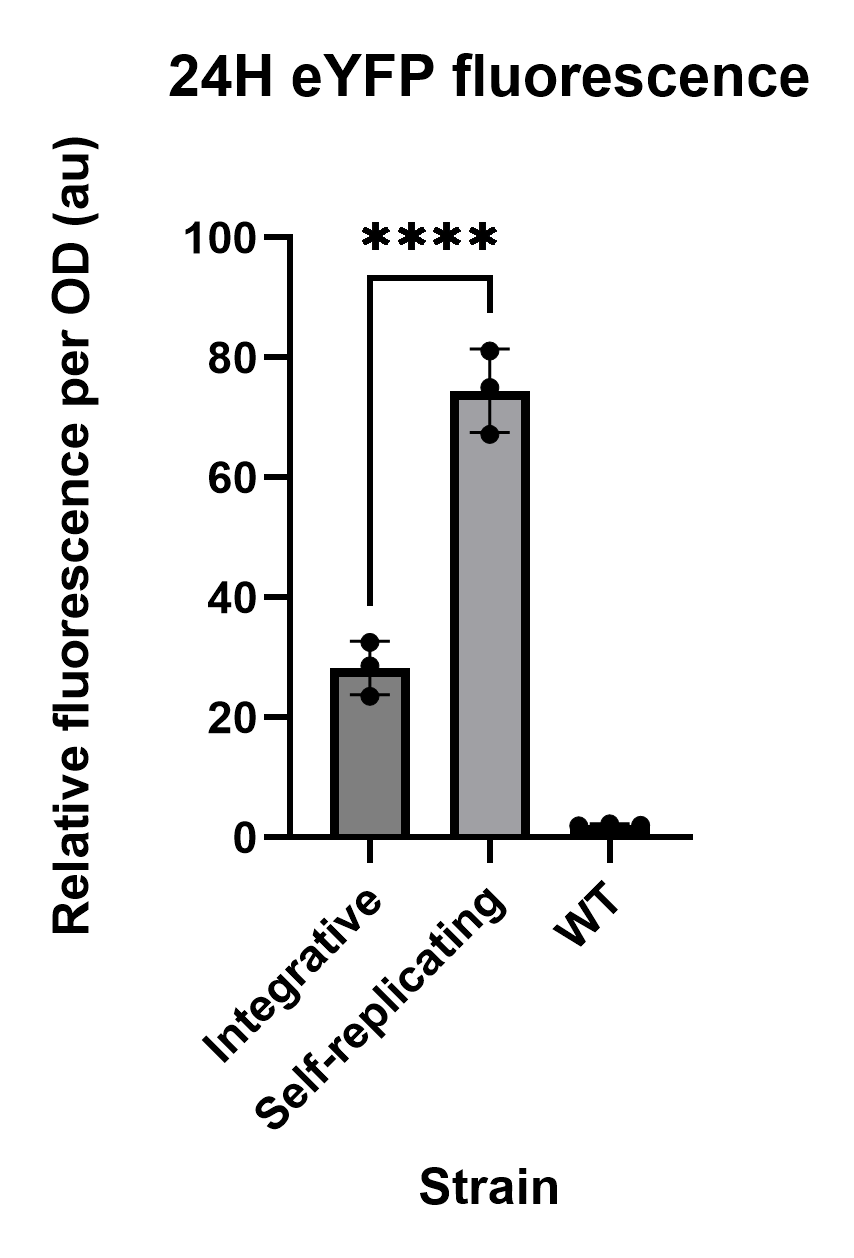

Supplement: Supplementary file 1 [file biomolecules-16-00870-s001.zip › Figure S10 - RSF1010 vs mrr under constitutive expression (Pcpc560, 24h).tif]

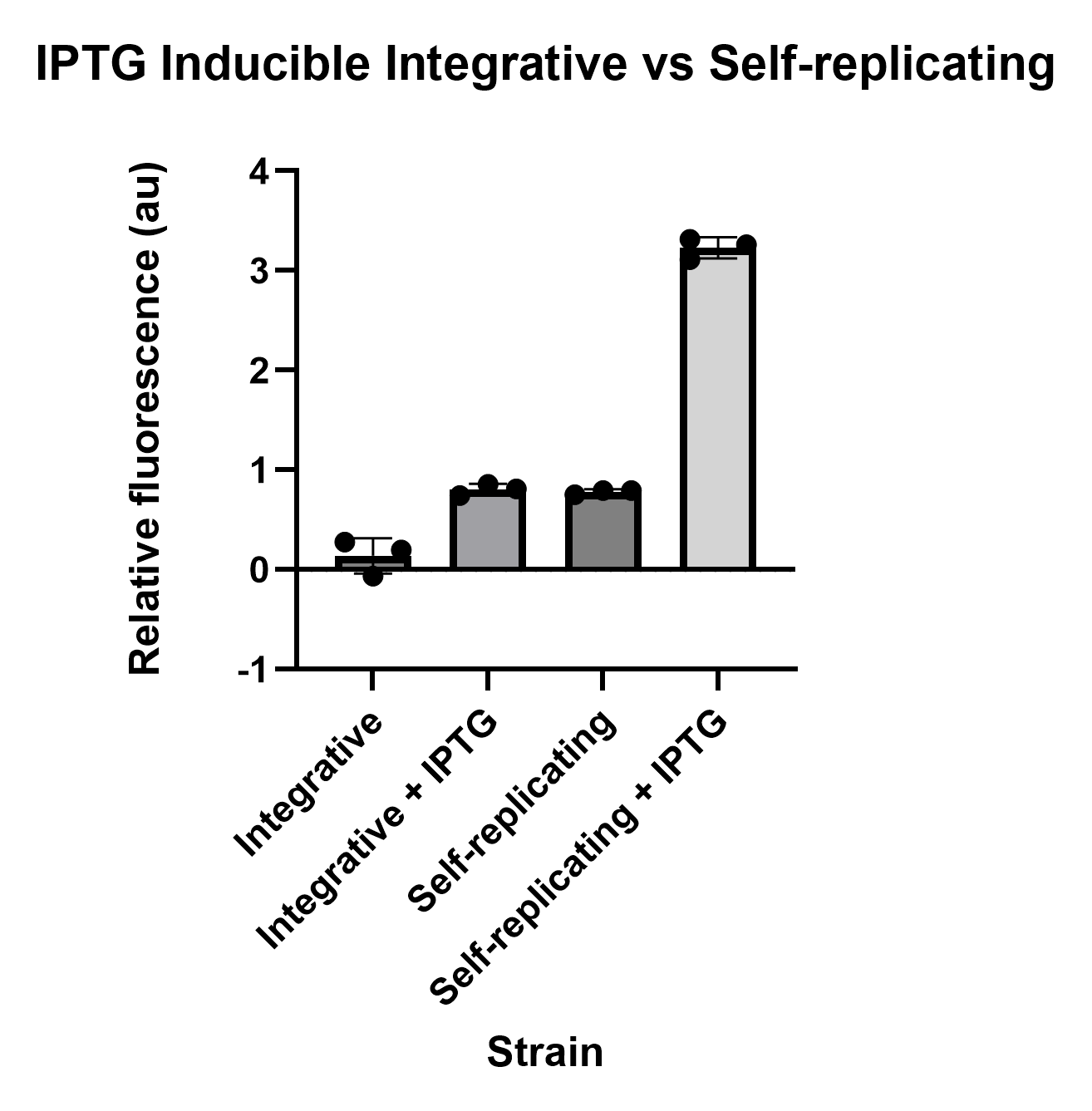

Supplement: Supplementary file 1 [file biomolecules-16-00870-s001.zip › Figure S11 - RSF1010 vs mrr under IPTG-inducible expression.tif]

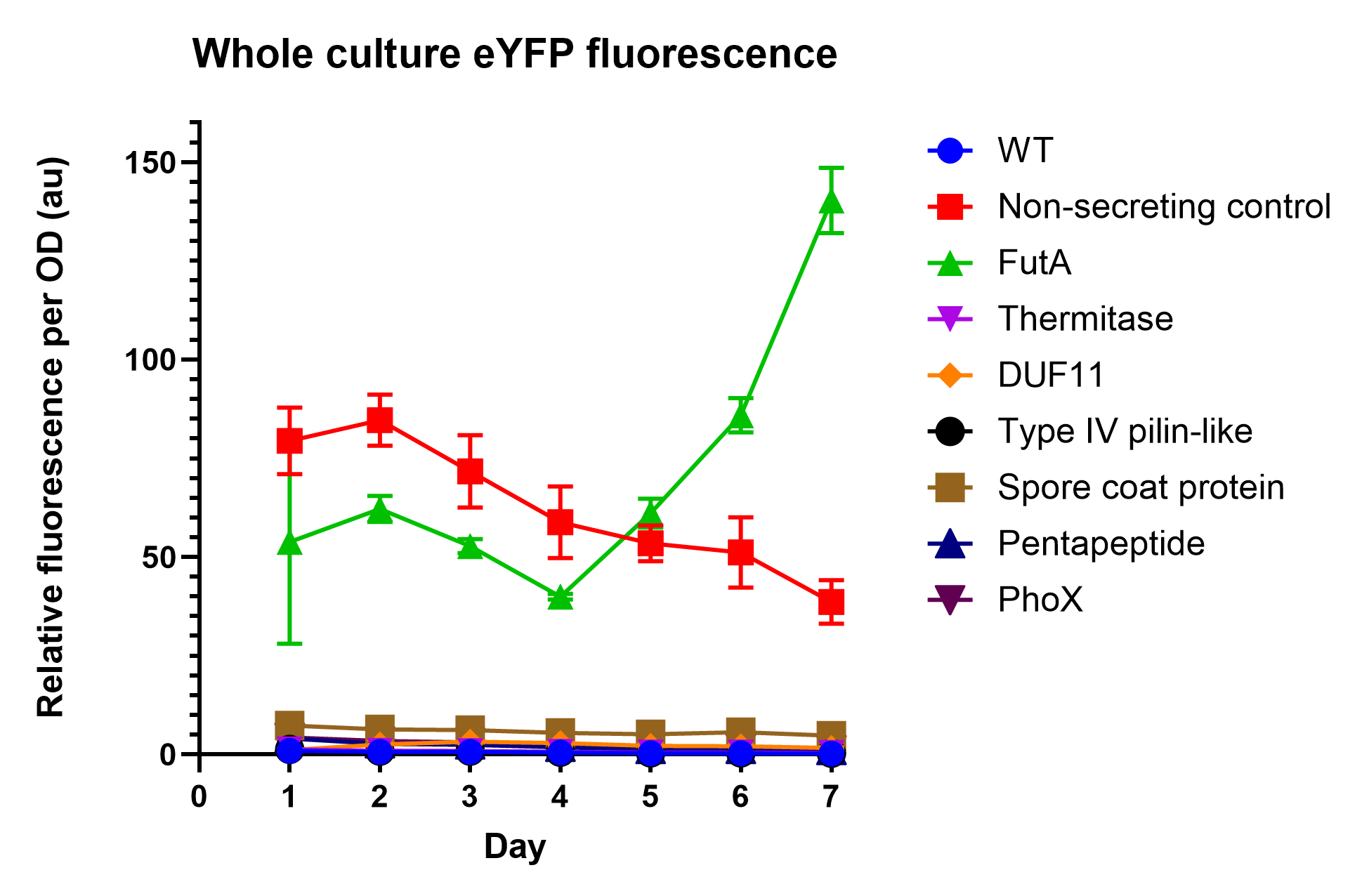

Supplement: Supplementary file 1 [file biomolecules-16-00870-s001.zip › Figure S2 Whole-culture eYFP fluorescence time course.tif]

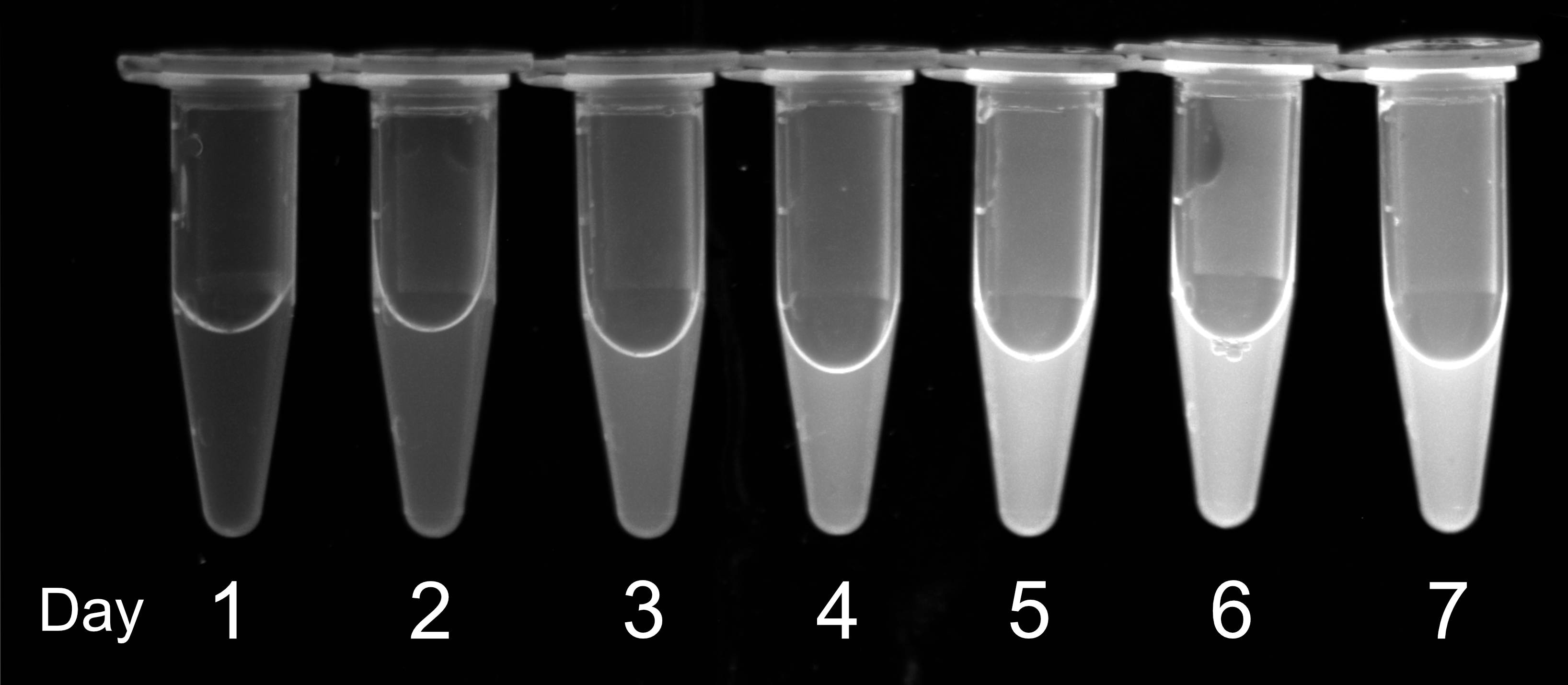

Supplement: Supplementary file 1 [file biomolecules-16-00870-s001.zip › Figure S3 Chemidoc UV Images futA fluorescence time course.tif]

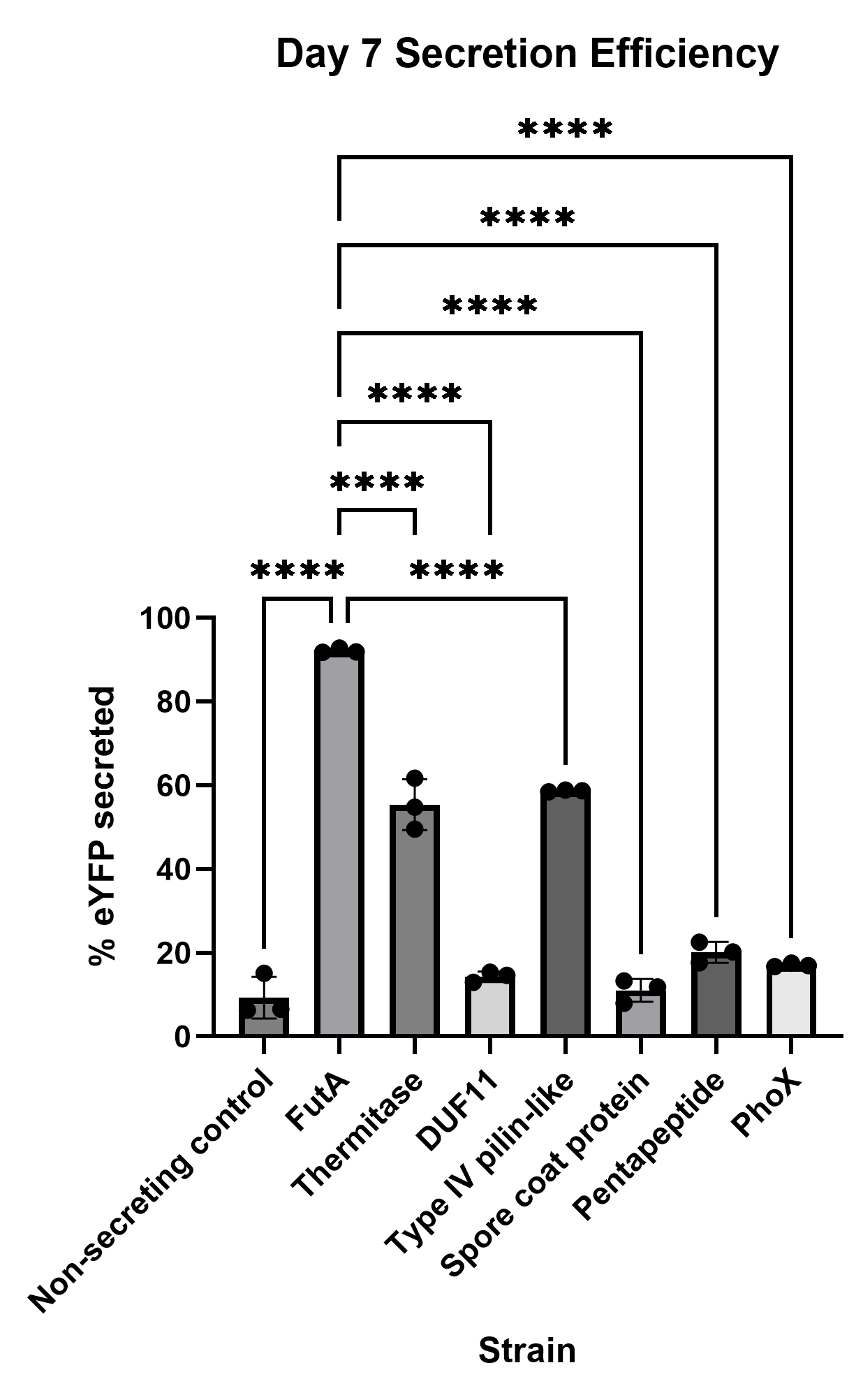

Supplement: Supplementary file 1 [file biomolecules-16-00870-s001.zip › Figure S4 % Secreted day 7 bart chart .tif]

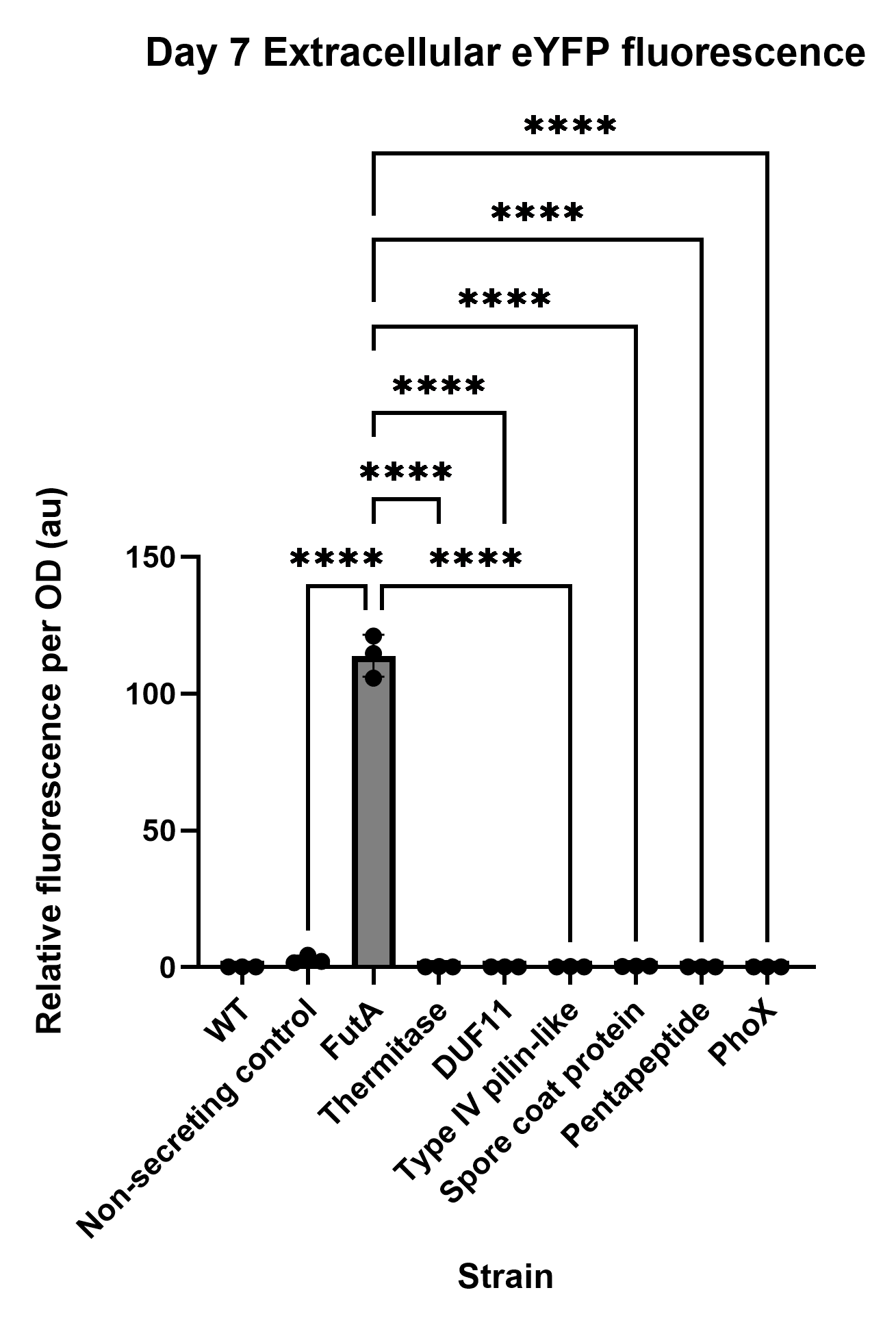

Supplement: Supplementary file 1 [file biomolecules-16-00870-s001.zip › Figure S5 Extracellular day 7 bar chart.tif]

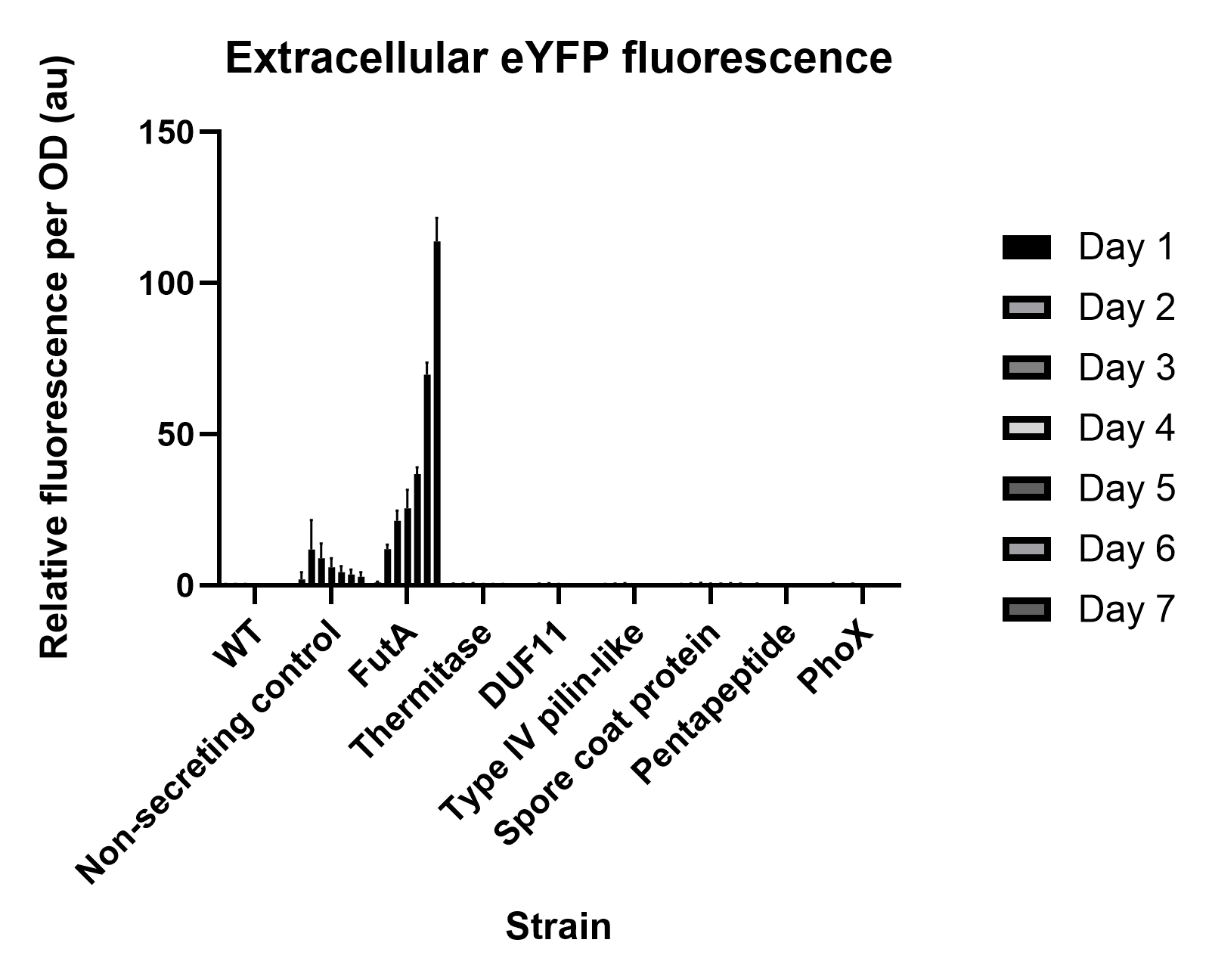

Supplement: Supplementary file 1 [file biomolecules-16-00870-s001.zip › Figure S6 Grouped daily extracellular eYFP fluorescence.tif]

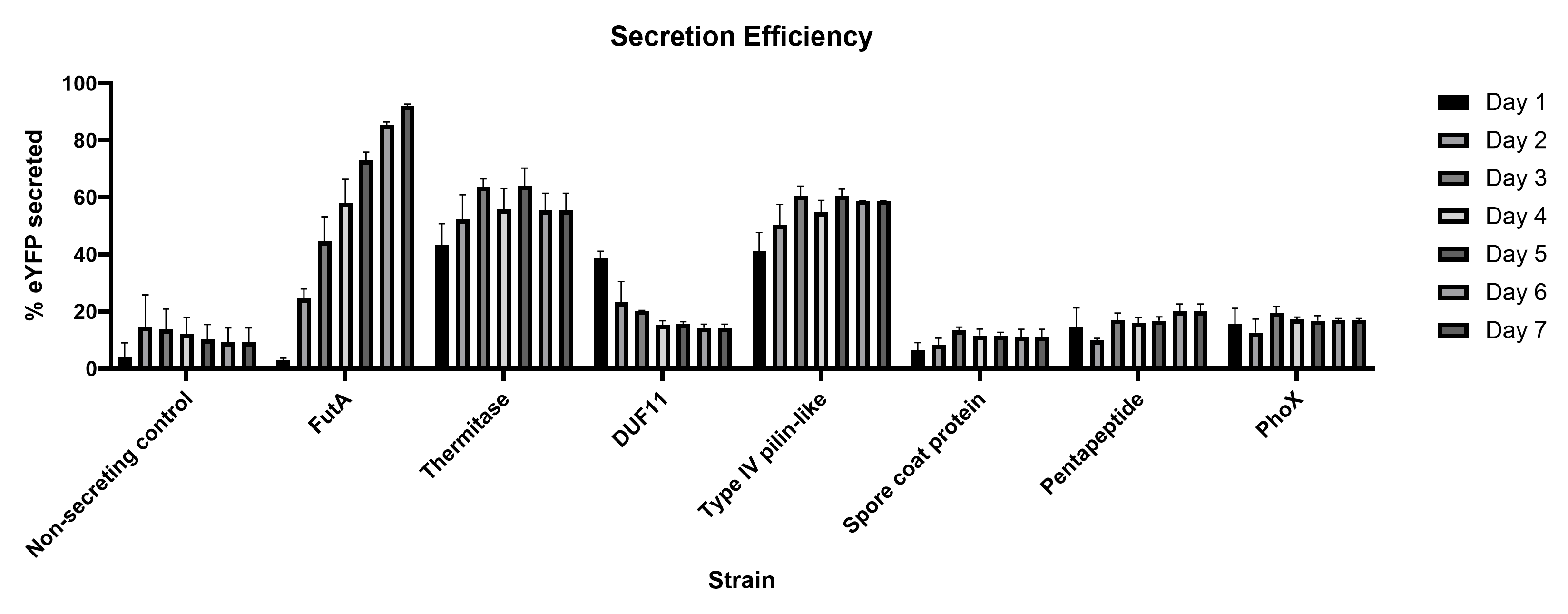

Supplement: Supplementary file 1 [file biomolecules-16-00870-s001.zip › Figure S7 - Grouped daily secretion efficiency for all constructs.tif]

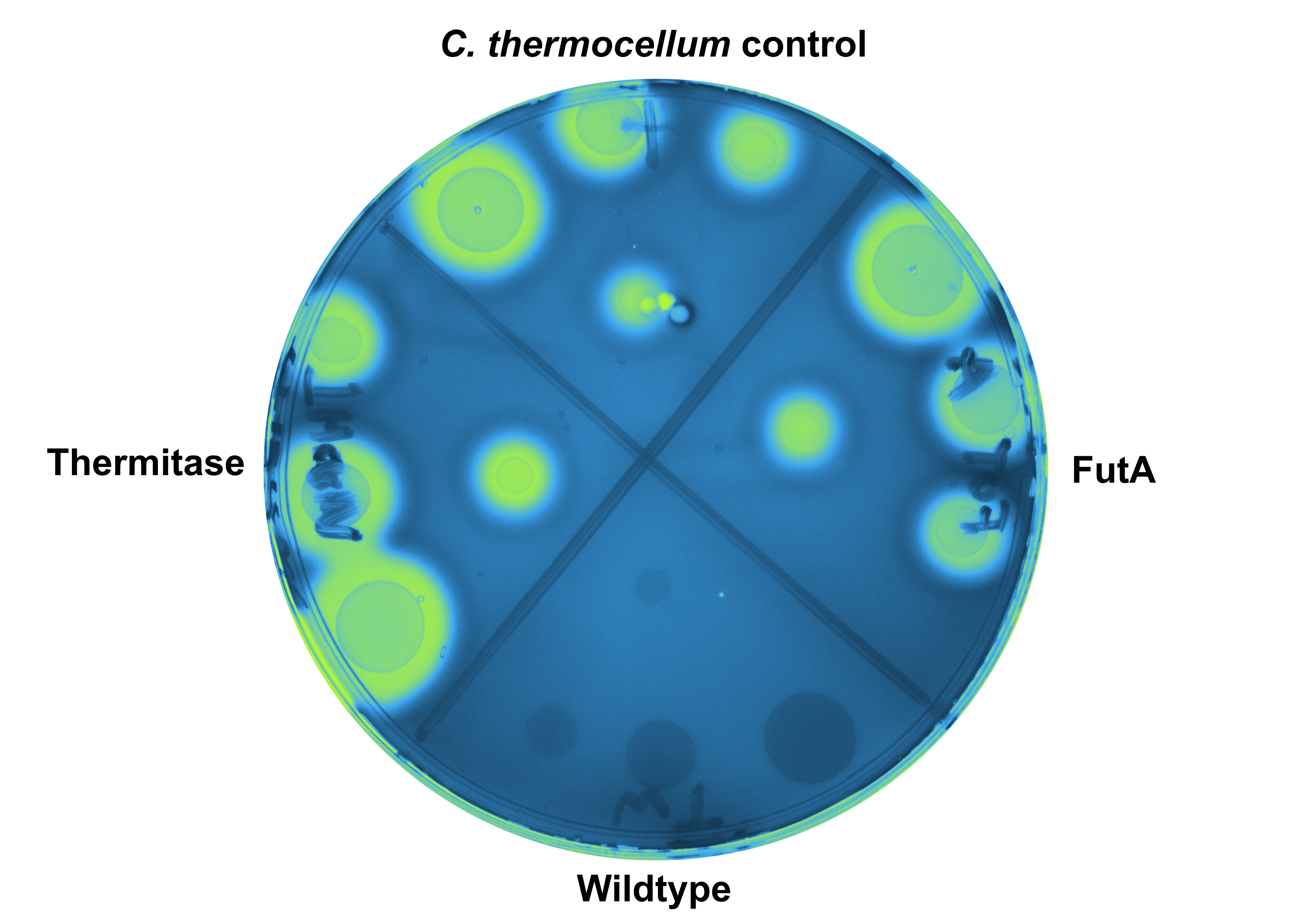

Supplement: Supplementary file 1 [file biomolecules-16-00870-s001.zip › Figure S8 Congo Red colony overlay assay.tif]

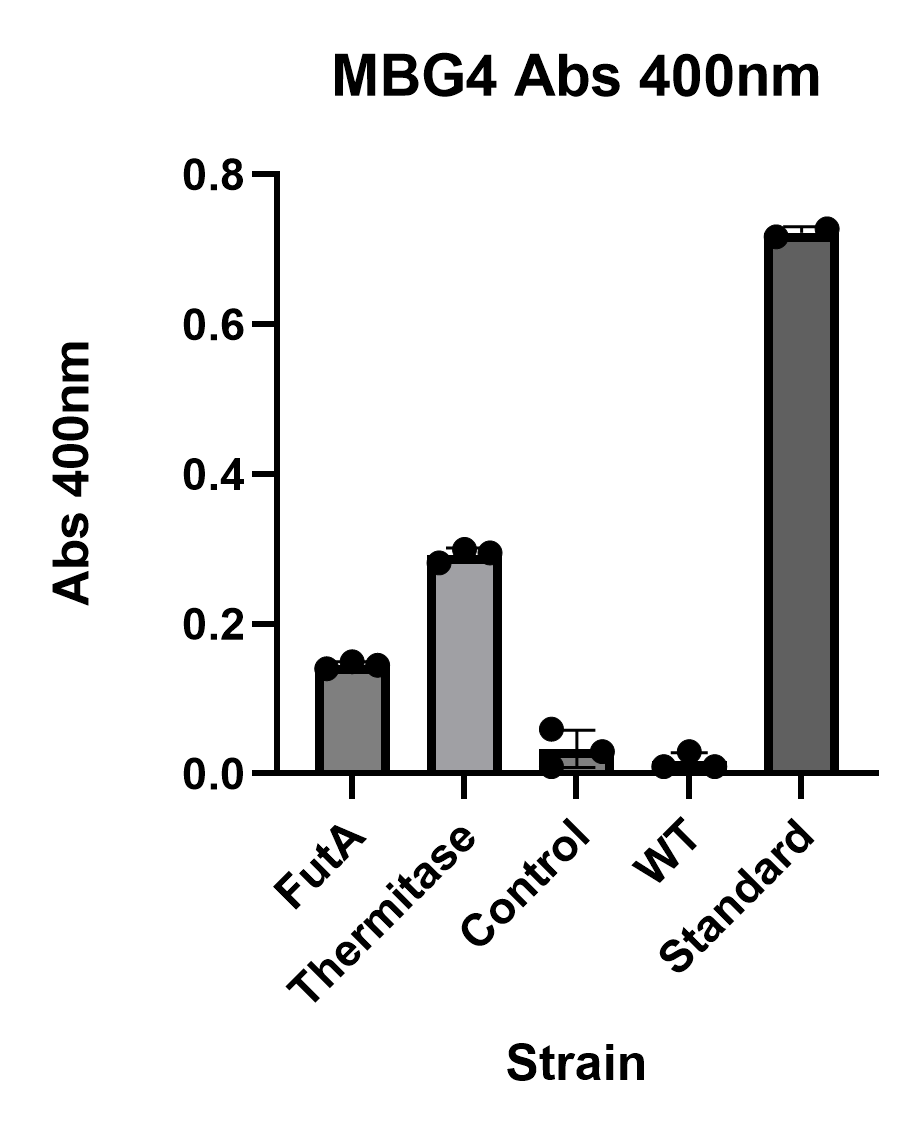

Supplement: Supplementary file 1 [file biomolecules-16-00870-s001.zip › Figure S9 - MBG4 raw Absorbance at 400nm.tif]
